# The effectiveness of a web-based information-knowledge-attitude-practice continuous intervention on the psychological status, medical compliance, and quality of life of patients after coronary artery bypass grafting surgery: a parallel randomized clinical trial

**DOI:** 10.1186/s13019-024-02618-w

**Published:** 2024-03-13

**Authors:** Jin Li, Yueli Deng, Yan Jiang

**Affiliations:** 1 Department of Emergency Medicine, Shengjing Hospital of China Medical University, No.36 Sanhao Street, Heping District, Shenyang, Liaoning Province 110004 China; 2 Department of Intensive Care Medicine, Shengjing Hospital of China Medical University, No.36 Sanhao Street, Heping District, Shenyang, Liaoning Province 110004 China

**Keywords:** Coronary artery bypass grafting, Information-knowledge-attitude-practice, Anxiety and depression, Quality of life, Major adverse cardiac and cerebrovascular events, Web-based nursing intervention

## Abstract

**Background:**

Coronary artery disease (CAD) patients who have undergone coronary artery bypass grafting (CABG) often experience a severe psychological burden for a long period of time, which can adversely affect their post-operative prognosis. Therefore, this study aimed to evaluate the effect of a web-based Information-Knowledge-Attitude-Practice (WIKAP) continuous intervention on the psychological status, medical compliance, and quality of life (QoL) in patients with CAD after CABG surgery.

**Methods:**

A parallel randomized clinical trial enrolled 174 CAD patients who underwent CABG at our hospital between January 2018 and December 2019. The participants were randomly divided into the Control and WIKAP group and received intervention for 12 months. The scores for anxiety, depression, medical compliance, and QoL were assessed on the first day (M0), 3rd month (M3), 6th month (M6), 9th month (M9) and 12th month (M12) after discharge. Furthermore, the occurrence of major adverse cardiac and cerebrovascular events (MACCE) was analyzed using the Kaplan-Meier curve and Cox proportional regression models for an additional 24-month follow-up period without any intervention.

**Results:**

After the 12-month intervention, the scores of anxiety and depression were significantly reduced in the WIKAP group at M9 and M12 compared to those in the Control group (all *P* < 0.05). Additionally, the scores of medical compliance in the WIKAP group were remarkably elevated at M6, M9, and M12 compared with those scores in the Control group (all *P* < 0.05). Furthermore, the QoL scores were lower in the WIKAP group at M6, M9, and M12 compared to the Control group (all *P* < 0.05). However, the MACCE-free survival showed no significant difference between the two groups (*P* > 0.05). Cox proportional regression analysis also showed that the nursing intervention (Control vs. WKIAP) was not associated with the incidence of MACCE.

**Conclusion:**

WIKAP nursing intervention effectively improved the psychological health, medical compliance, and QoL in CAD patients who underwent CABG operation, but it did not prolong MACCE-free survival.

**Trail registration:**

The study is registered in isrctn.org: ISRCTN13653455.

## Introduction

Coronary artery disease (CAD), one of the most commonly diagnosed cardiovascular diseases (CVD), has been rapidly increasing among the Chinese population in recent years [1, 2]. A recent report has confirmed that nearly 11 million patients with CVD in China benefit from coronary artery bypass grafting (CABG) operations throughout the country [3], which demonstrated effective efforts to improve symptoms of coronary artery lesions, restore blood supply, prevent myocardial infarction, and increase patients’ chances of survival [4]. Nevertheless, due to the inevitable pathological and physiological dysfunctions caused by the CABG postoperative procedure, it takes a long time for CAD patients to recover, especially after they are discharged from the hospital [5]. For instance, prior literature has illustrated that during the lengthy and uncertain rehabilitation period, a majority of patients who undergo CABG surgery experience symptoms such as fatigue, sleeplessness, fear, irritability, anxiety, and depression. These symptoms can directly impact the poor prognosis and increase the risk of major adverse cardiac and cerebrovascular events (MACCE) among patients with CAD [6]. Therefore, helping postoperative CABG patients improve their rehabilitation process and reduce the risk of CAD recurrence is considered a challenging task for medical staff to overcome.

Accumulating evidence has demonstrated that continuous nursing could effectively meet the needs of CVD patients and their caregivers by providing prolonged high-quality medical services for cardiac rehabilitation [7]. Currently, the Information-Knowledge-Attitude-Practice (IKAP) theory, regarded as a relational nursing model, has been applied in clinical nursing care work and has achieved prominent effects on patients with various disorders, such as chronic obstructive pulmonary disease [8], cerebrovascular disease [9], and gastric cancer [10]. According to the IKAP theory, it requires hierarchical connections of four continuous progressions: “Information” and “Knowledge” are the fundamental steps to guarantee the alternation of a patient’s improper health-related behaviors. It allows medical staff to instruct patients in sufficiently grasping disease-related knowledge and strengthening their awareness by providing them with professional and systematic education. Meanwhile, “Attitude” and “Practice” are referred to as the driving forces that guide patients to improve their positive attitudes and self-care abilities to solve the difficulties they face during the rehabilitation period [11, 12]. However, little is known about the efficacy of the IKAP continuous nursing model in improving the recovery of CAD patients after CABG surgery.

Based on the rapid development of Internet technology in China over the past few years, it has become very convenient for nursing providers to communicate with their patients at any time using digital tools [13]. Therefore, the aim of this study was to establish a web-based IKAP continuous intervention model (WIKAP) for CAD patients after CABG surgery and evaluate its application value, which might provide evidence for the clinical guidance of post-operative management for CABG patients.

## Materials and methods

### Study design and participants

The clinical data of 174 patients who underwent CABG surgery at Shengjing Hospital Affiliated to China Medical University (Shenyang, China) from January 10th, 2018, to December 10th, 2019, were enrolled into this study. Firstly, the eligible participants were randomly divided into two groups: the Control group (*n* = 87) and the WIKAP group (*n* = 87). The inclusion criteria were as follows: (1) diagnosed with CAD and underwent CABG surgery for the first time; (2) patients or their direct caregivers were capable of using web-based social media platforms (such as WeChat and QQ); (3) age > 18 years old; (4) in good mental condition and possess proper communication abilities; (5) willing to participate in this study voluntarily and sign the written informed consent. The exclusion criteria were as follows: (1) had cognitive or psychiatric disorders; (2) combined with other severe organ dysfunctional disorders; (3) unwilling to cooperate with researchers for any reason or refusal of receiving care intervention; (4) lack of vision, speaking, reading, or writing abilities; (5) experienced acute psychological or physical incidents; (6) died after the CABG operation; (7) a history of myocardial infarction or previous revascularization. The general demographic information was collected from each individual during hospitalization.

### Intervention methods

#### Conventional nursing care intervention

Before discharge, the routine nursing procedure was performed on patients in the Control group, which allowed responsible nursing staff to provide face-to-face oral education to patients and their caregivers regarding health knowledge about CABG operation programs, diet, medication, psychological consultation, and postoperative attention to complications. This education was given only once. Furthermore, regular follow-up was conducted via telephone every month after discharge. During these follow-up calls, attending nurses provided verbal reminders to patients about routine nursing education.

#### WIKAP intervention group

Besides providing routine nursing guidance, the participants in the WIKAP group received continuous nursing intervention based on the IKAP theory through the Internet. Primarily, a WIKAP continuous nursing team was established, comprising one emergency medicine physician, one cardiovascular physician, one psychologist, one head nurse, and four senior nurses. They were responsible for designing nursing plan, implementing it, and collecting and analyzing follow-up data. Subsequently, the team members were trained to master the methods and skills of implementing WIKAP continuous nursing in patients who underwent CABG surgery. The WIKAP continuous nursing intervention was implemented and lasted for 12 months after discharge. The procedures were listed as follows (details in Table 1):

**Table 1:** The procedures for implementing the WIKAP nursing program among CAD patients after CABG surgery

Procedures	Contents
**Information** **collection**	• WIKAP nursing team: Create a professional WIKAP continuous nursing team comprising of one emergency medicine physician, one cardiovascular physician, one psychologist, one head nurse, and four senior nurses. This team will be responsible for planning, designing, implementing, and analyzing follow-up data. • Health record establishment: Collect each patient’s health record before discharge: including personal data, such as age, gender, disease condition, disease-related knowledge, medication, diet, and psychological status. • WIKAP intervention implementation: Design a targeted WIKAP nursing program based on each patient’s specific needs and implement it immediately after discharge.
**Knowledge** **education**	• “WeChat and QQ”^a^ Nursing Group establishment: All participants are invited to join the “CABG Recovery Experience Share and Exchange Family” nursing groups on QQ and WeChat for online communication and follow-up. • Knowledge education program: CAD-related knowledge is sent to participants via the WeChat and QQ nursing group at 17: 00 PM every Tuesday and Friday. The updated information includes a basic understanding of coronary disease, guidance on medication usage, precautions regarding potential possible adverse effects, appropriate rehabilitation exercise, daily self-care techniques, dietary interventions, and psychological management. • Our nursing team experts conducted regular educational seminars to encourage patients and their caregivers to attend online meeting and learn professional knowledge about CABG rehabilitation. • Response to patients’ questions: Our nursing staff will be responsible for answering the online questions they ask at 15:00 PM every day.
**Attitudes generation**	• Weekly video follow-up: One-to-one weekly video follow-up sessions are conducted after discharge to track the participants’ recovery progress and performance. For patients with poor compliance behavior, one-to-one education is needed to help them understand the potential adverse consequences of incorrect behaviors. • Remind caregivers to constantly consider the patient’s health-related behaviors and instruct them on how to encourage patients to build their confidence. • Successful recovery experience sharing: In order to help patients regain their confidence, we invite previous patients who have successfully recovered to share their self-care experiences via WeChat and Tencent QQ groups every Sunday at 19:00 PM. • Establishment of a stable nurse-patient relationship: Encourage patients to share their inner feelings and provide targeted psychological guidance every Sunday at 19:00 PM.
**Practice formation**	• Remind patients to take their daily medication at 9:00 AM every day. • Monitor each patient’s recovery process: All patients are encouraged to record their daily diet, activities, and physical reactions in the WeChat and Tencent QQ group. • Update self-care plan: The responding nurses constantly adjust the nursing plan to better suit each patient’s condition based on their daily behavioral performance and records during the weekly follow-up visits. • Patients’ compliance improvement: If the patients do not adhere to the care plan (including diet, medication, exercise, and psychological well-being), the nursing staff will assist them in promptly modifying their daily behaviors. These patients will be critically focused on by our nursing staff to ensure that their desired goals are achieved during the next follow-up visit.

**Notes**: CABG, coronary artery bypass grafting; CAD, coronary heart disease; WIKAP, web-based Information-Knowledge-Attitude-Practice. ^a^WeChat and Tencent QQ software: the most popular social media application in China

### Outcome measurements

#### Depression and anxiety evaluation

The Hospital Anxiety and Depression Scale (HADS), originally developed by Zingmod and Snaith, is widely used to measure anxiety and depression states among various populations in hospital and outpatient clinic settings [14]. The Chinese version of the self-administered HADS was validated for good reliability and validity by previous researchers in Chinese patients with CAD [15, 16]. The HADS includes two subscales: HADS-A for anxiety assessment and HADS-D for depression assessment. Both HADS-A and HADS-D are rated on a 4-point Likert scale and includes 7 scoring items. The total score ranges from 0 to 21, with score of 0–7 indicating no depression or anxiety, 8–10 indicating mild depression or anxiety, 11–14 indicating moderate depression or anxiety, and 15–21 indicating severe depression or anxiety. If the score of HADS-A or HADS-D > 7, it is considered as anxiety or depression.

#### Medication compliance

The patient’s medication compliance was assessed using the Chinese version of Morisky’s Medication Adherence Scale (MMAS-8) (Reliability and validity of a modified 8-item Morisky Medication Adherence Scale in patients with chronic pain) [17]. The scale consists of 8 items that requires a “Yes” or “No” response. The total score was 8 points, of which a score < 6 indicated low compliance, a score between 6 and 7 indicated medium compliance, and a score = 8 indicated high compliance.

#### Quality of life (QoL) measurement

The QoL was measured using the Chinese version of the 12-Item Short-Form Health Survey (SF-12), a shorter version of the previous Short Form 36 Health Survey (SF-36) [18]. The SF-12 consists of 2 subscales: the Physical Component Summary (PCS) and the Mental Component Summary (MCS) to determine the physical and emotional health status, respectively. Each subscale contains 12 items, with a total score ranging from 1 to 100. Higher scores indicated better health-related QoL among the participants.

#### Follow-up for MACCE assessment

After the 12-month intervention period, a subsequent follow-up period without any intervention was conducted for an additional 24 months (total 36-month follow-up) through telephone contact or hospital readmission to monitor the occurrence of the MACCE events. The occurrence of MACCE among these CAD patients was defined as the complex of repeat revascularization, stroke, myocardial infarction, and death according to the clinical diagnoses [19].

### Data collection

During the 12-month intervention follow-up period, the scores for anxiety, depression, MMAS-8, and QoL were assessed on the first day (M0), 3rd month (M3), 6th month (M6), 9th month (M9) and 12th month (M12) after discharge. The rate of MACCE occurrence was recorded throughout the entire 36-month follow-up period.

### Sample size

PASS V11.0 software (NCSS, LLC, USA) was used to calculate the sample size in this study. According to a previous report [15], the sample size was estimated based on the predetermined anxiety/depression rates at M12: 35% in the Control group and 15% in the WIKAP group, respectively. Based on the power of variation (1-β) at 80%, a two-sided significance level (α) of 5%, and accounting for a minimum attrition rate of 20%, it was necessary to recruit 87 CAD patients who had undergone CABG operation in each group.

### Statistical analysis

On the basis of the intention-to-treat (ITT) principle [20], all 174 participants were included in the final analyses, and the last observation carried forward (LOCF) method was used to handle the missing data. The statistical data were analyzed and graphed using SPSS statistical analysis software version 26.0 (IBM Corp., NY, USA) and GraphPad Prism software version 7.01 (GraphPad Software Inc., Chicago, USA). The continuous data were verified to conform to the normal distribution and described as mean ± standard deviation (SD). The categorical data were expressed as numbers (n) and percentages (%). A Chi-square test, Student’s *t*-test, or Wilcoxon rank-sum test was used to compare the two groups. Kaplan-Meier curve was used to describe the MACCE-free survival during the 36-month follow-up period, and the difference in the survival rate between the two groups was calculated using the log-rank test. Univariate and multivariate Cox proportional hazard regression models were performed to analyze the nursing interventions associated with the occurrence of MACCE. *P* < 0.05 indicated statistically significant.

## Results

### Study flow

**Fig. 1:**
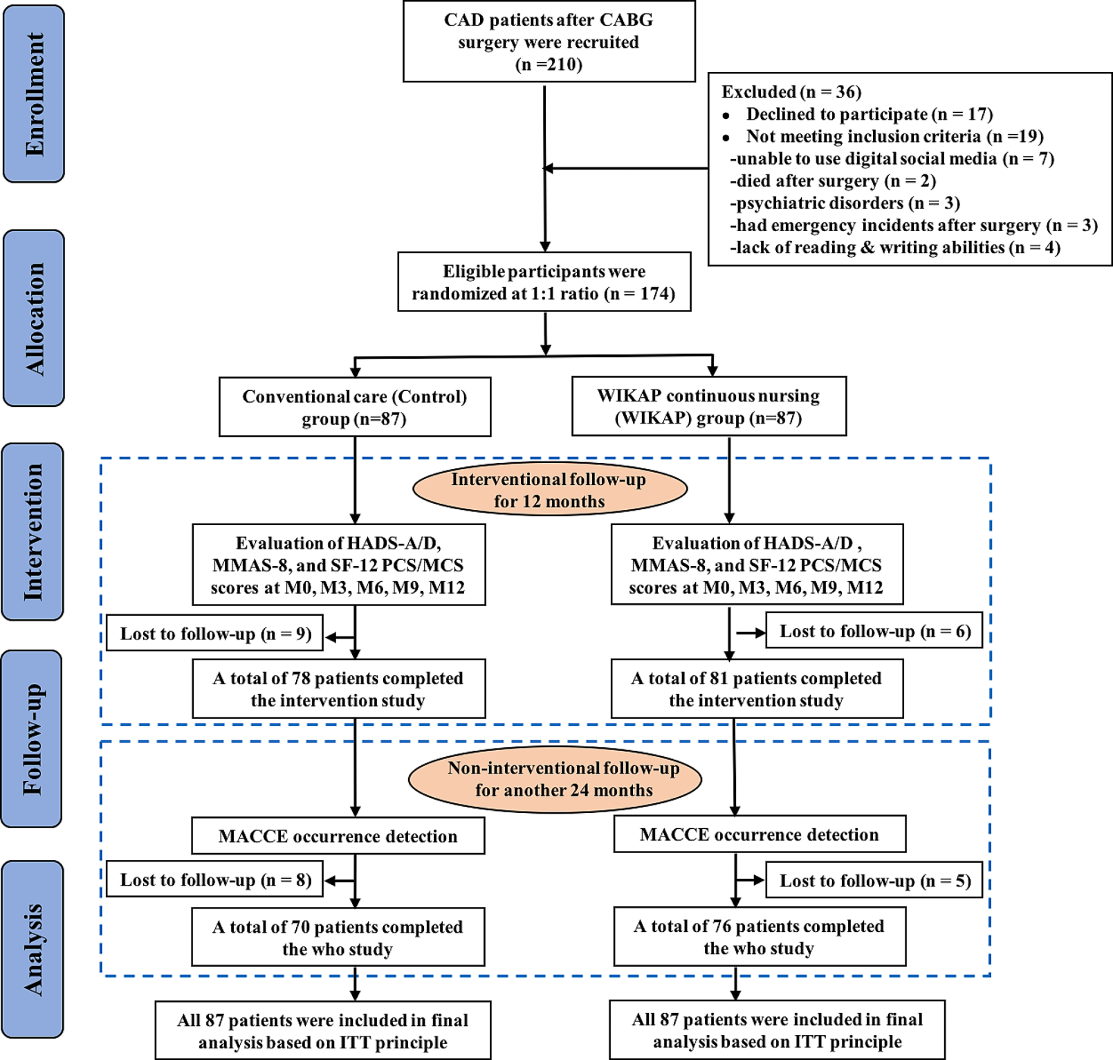
Study flow diagram

As shown in Fig. 1, a total of 210 CAD patients who underwent CABG operation were initially recruited. However, 36 cases were excluded due to their unqualified conditions. Eventually, data from 174 patients in both groups were collected until the end of the analysis according to the ITT principle. As shown in Table 2, the average age of patients in the Control group was 60.80 ± 9.39 years old (range 42–75), with a female/male ratio of 36/51, while patients in the WIKAP group had an average age of 61.09 ± 8.08 years old (range 42–77) and a female/male ratio of 39/48. No significant difference was found among the sociodemographic characteristics, such as age, gender, body mass index (BMI), education, annual household income, employment, marital status, and residence, between the Control group and WIKAP group (all *P* > 0.05, Table 2). For clinical parameters, the most common risk factors for CAD in the Control group were hypertension (56.32%), followed by a history of smoking (43.68%) and hyperlipidemia (40.23%); while in the WIKAP group, the most common risk factors were a history of smoking (55.17%), hyperlipidemia (51.72%), and hypertension (49.43%), respectively. However, there were no significant differences in CAD risk factors, New York Heart Association (NYHA) classification, lesion vessels, and left ventricular ejection fraction (LVEF) between the two groups at baseline (all *P* > 0.05, Table 2).

**Table 2:** Comparison of baseline characteristic of CAD patients undergone CABG operation between the two groups (*n* = 174)

Items	Control group (*n* = 87)	WIKAP group (*n* = 87)	t/x^2^	*P*
Age (years), Mean ± SD	60.80 ± 9.39	61.09 ± 8.08	0.283	0.778
Gender, No. (%)				
Female	36 (41.38)	39 (44.83)	0.211	0.646
Male	51(58.62)	48 (55.17)		
BMI (kg/m^2^), Mean ± SD	23.62 ± 2.05	24.09 ± 2.09	1.505	0.134
Education level, No. (%)				
Middle school or less	57 (65.52)	49 (56.32)	1.545	0.214
High school or above	30 (34.48)	38 (43.68)		
Marital status				
Married	59 (67.82)	68 (78.16)	2.361	0.124
Single/Widowed/Divorced	28 (32.18)	19 (21.84)		
Employment status, No. (%)				
Employed	34 (39.08)	41 (47.13)	1.148	0.284
Unemployed	53 (60.92)	46 (52.87)		
Annual household income, No. (%)				
&lt; 30,000	19 (21.84)	23 (26.44)	1.616	0.446
30,000–50,000	36 (41.38)	28 (32.18)		
&gt;50,000	32 (36.78)	36 (41.38)		
Living location, No. (%)				
Urban	46 (52.87)	51 (68.62)	0.582	0.445
Rural	41 (47.13)	36 (41.38)		
NYHA classification, No. (%)				
I-II	58 (66.67)	52 (59.77)	0.890	0.346
III	29 (33.33)	35 (40.23)		
Lesion vessels, No. (%)				
1	5 (5.75)	4 (4.60)	0.861	0.650
2	48 (55.17)	54 (62.07)		
≥3	34 (39.08)	29 (33.33)		
CAD risk factors, No (%)				
Hypertension	49 (56.32)	43 (49.43)	0.830	0.362
Hyperlipidemia^a^	35 (40.23)	45 (51.72)	2.314	0.128
Diabetes	28 (32.18)	20 (22.99)	1.841	0.175
Smoker history	38 (43.68)	48 (55.17)	2.299	0.129
Family history of CAD	19 (21.84)	12 (13.79)	1.923	0.166
LVEF, No. (%)				
&lt;50%	30 (34.48)	24 (27.59)	0.967	0.326
≥50%	57 (65.52)	63 (72.41)		

**Notes**: Statistical analysis was calculated by Student’s *t*-test or Chi-square test. BMI, body mass index; CABG, coronary artery bypass grafting; CAD, coronary heart disease; LVEF, left ventricular ejection fraction; NYHA, New York heart association; SD, standard deviation; WIKAP, web-based Information-Knowledge-Attitude-Practice. ^a^Hyperlipidemia was defined as total cholesterol ≥ 6.22 mmol/L, low-density lipoprotein cholesterol > 4.14 mmol/L, high-density lipoprotein cholesterol < 1.04 mmol/L, or triglyceride ≥ 2.26 mmol/L

### The effect of web-based IKAP intervention on anxiety

**Fig. 2:**
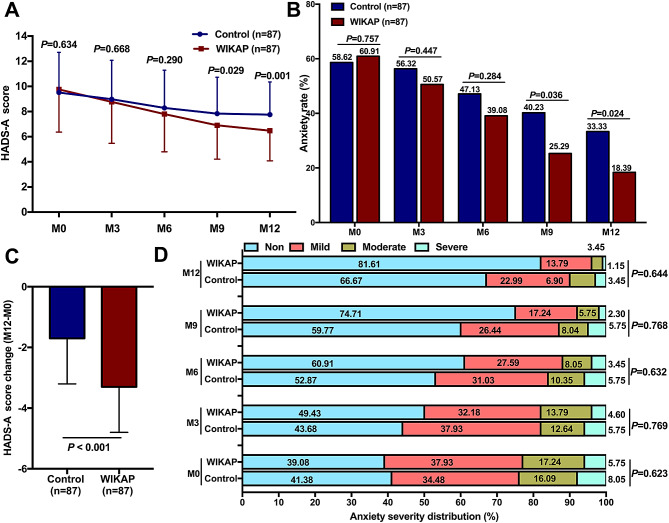
The effect of WIKAP nursing on anxiety in CAD patients with CABG post-operation. **A** The comparison of HADS-A score, **B** Anxiety rate, **C** HADS-A score change (M12-M0), and **D** The distribution of anxiety severity between the Control and WIKAP groups at M0, M3, M6, M9, and M12 post-intervention. *P* < 0.05 was considered statistically significant

There was no significant difference in HADS-A scores between the Control group and the WIKAP group at M0 (*P* = 0.634), M3 (*P* = 0.668), or M6 (*P* = 0.290) time points (Fig. 2A). However, the HADS-A scores were significantly reduced in the WIKAP group at M9 (*P* = 0.029) and M12 (*P* = 0.001) compared to the Control group (Fig. 2A). The results also showed that the anxiety rate was similar between the two groups at M0 (*P* = 0.757), M3 (*P* = 0.447), and M6 (*P* = 0.284) (Fig. 2B). However, the number of anxious patients was significantly lower in the WIKAP group at M9 (*P* = 0.036) and M12 (*P* = 0.024) compared to those in the Control group (Fig. 2B). Furthermore, the change in HADS-A score (M12-M0) was more pronounced in the WIKAP group than that in the Control group (*P  *< 0.001, Fig. 2C). However, no significant difference in anxiety severities was found at M0 (*P* = 0.623), M3 (*P* = 0.769), M6 (*P* = 0.632), M9 (0.768), or M12 (*P* = 0.644) between the two groups (Fig. 2D).

### The influence of web-based IKAP intervention on depression

**Fig. 3:**
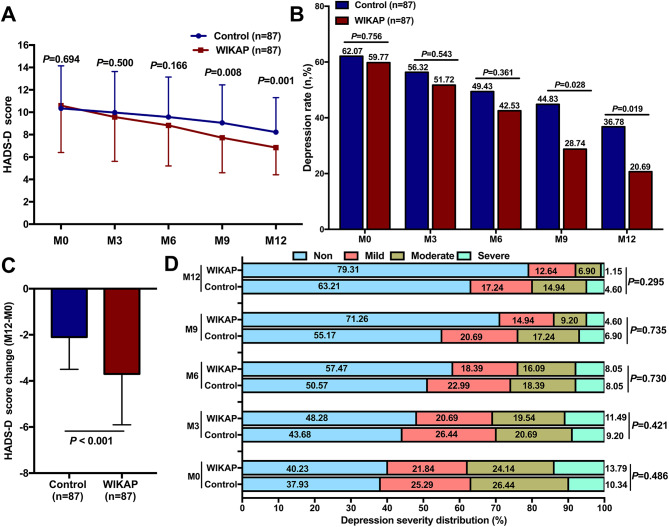
The influence of WIKAP nursing on depression in CAD patients with CABG post-operation. **A** The comparison of HADS-D score, **B** Depression rate, **C** HADS-D score change (M12-M0), and **D** The distribution of depression severity between the Control and WIKAP groups at M0, M3, M6, M9, and M12. *P* < 0.05 was considered statistically significant

As shown in Fig. 3A, similar HADS-D scores were obtained from patients in the both groups at M0 (*P* = 0.694), M3 (*P* = 0.500), or M6 (*P* = 0.166) during the 12-month follow-up (Fig. 3A). However, the HADS-D scores were significantly lower in the WIAKP group compared to the Control group at M9 (*P* = 0.008) and M12 (*P* = 0.001) post-intervention (*P *< 0.05, respectively, Fig. 3A). Moreover, there was no significant difference in the depression rate between the two groups at M0 (*P* = 0.756), M3 (*P* = 0.543), or M6 (*P* = 0.361) (Fig. 3B). However, at M9 and M12 post-intervention, the depression rates were significantly lower in the WIKAP group compared to those in the Control group (*P* = 0.028 and *P* = 0.019, respectively, Fig. 3B). Additionally, the patients in the WIKAP group showed an obviously change in HADS-D score (M12-M0) compared with that in the Control group (*P  *< 0.001, Fig. 3C). As for the detection of depression severity, the results revealed that the severity of depression in the WIKAP group were similar to those in the Control group at M0 (*P* = 0.486), M3 (*P* = 0.421), M6 (*P* = 0.730), M9 (*P* = 0.735), and M12 (*P* = 0.295) time points (Fig. 3D).

### The effect of web-based IKAP intervention on medication compliance

**Fig. 4:**
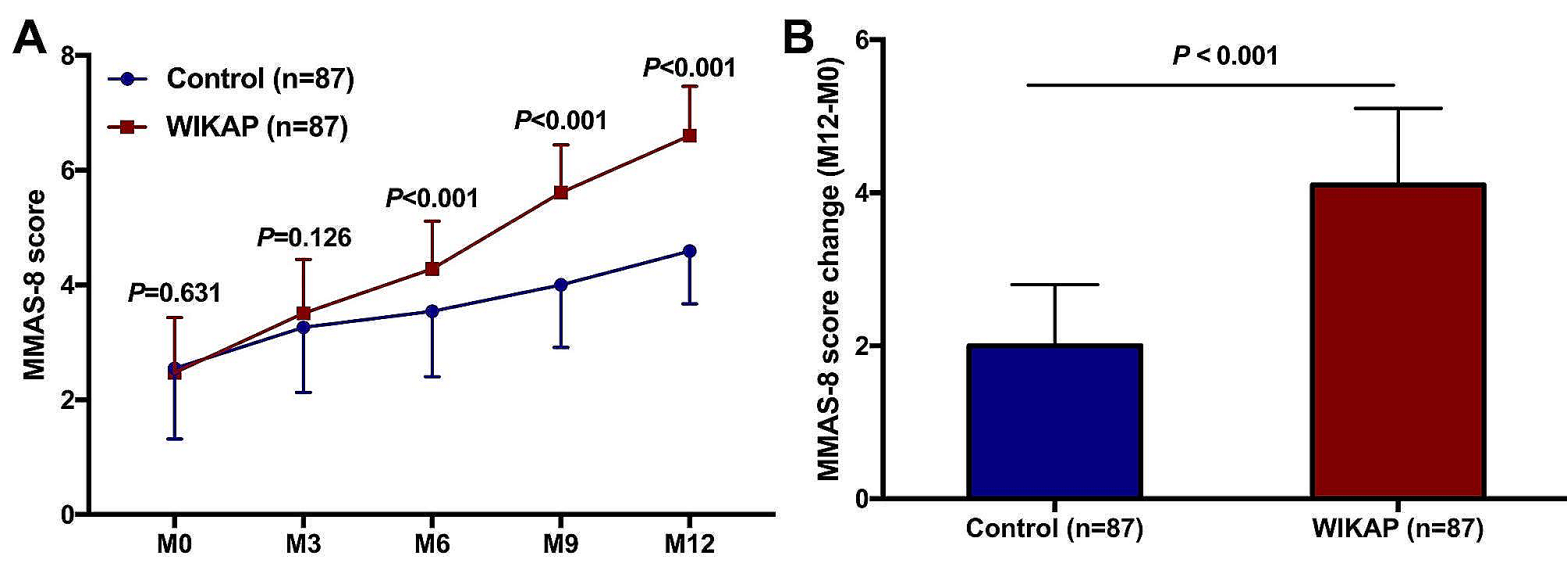
The effect of WIKAP nursing on medication compliance in CAD patients with CABG post-operation. **A** The comparison of MMAS-8 score and **B** MMAS-8 score change (M12-M0) between the Control and WIKAP groups at M0, M3, M6, M9, and M12. *P* < 0.05 was considered statistically significant

During the 12-month intervention period, although the temporal trend curve of MMAS-8 showed that there was no significant difference in medication compliance between the Control and WIKAP groups at M0 (*P* = 0.631) and M3 (*P* = 0.126) (Fig. 4A), the MMAS-8 scores gradually improved in both groups. Patients from the WIKAP group showed significantly elevated MMAS-8 scores at M6 (*P  *< 0.001), M9 (*P  *< 0.001), and M12 (*P  *< 0.001) compared to the corresponding scores in the Control group (Fig. 4A). Moreover, the change of the MMAS-8 score (M12-M0) in the WIKAP group was apparently higher than that in the Control group after 12 months post-intervention (*P  *< 0.001, Fig. 4B).

### The impact of web-based IKAP nursing on QoL

**Fig. 5:**
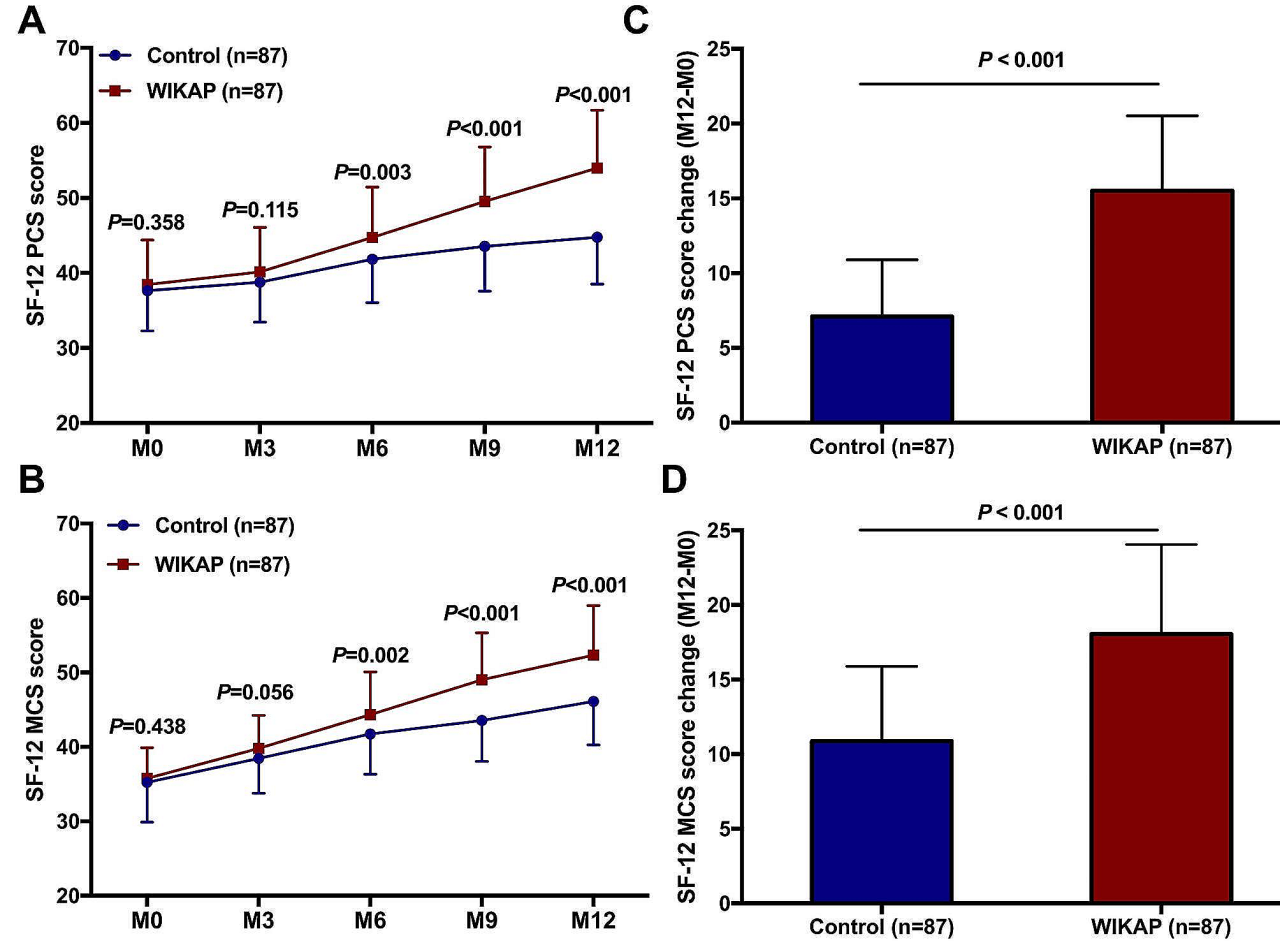
The impact of WIKAP nursing on QoL in CAD patients with CABG post-operation. **A** The comparison of SF-12 PCS score and **B** SF-12 MCS score between the Control and WIKAP groups at M0, M3, M6, M9, and M12. **C** The comparison of SF-12 PCS score change (M12-M0) and **D** SF-12 MCS score change (M12-M0) between the Control and WIKAP groups. *P* < 0.05 was considered statistically significant

The SF-12 PCS scores of patients at M0 (*P  *= 0.358) and M3 (*P  =* 0.115) were similar between the Control group and WIKAP group (Fig. 5A). However, the SF-12 PSC scores were apparently increased in the WIKAP group at M6 (*P  *= 0.003), M9 (*P  *< 0.001) and M12 (*P  *< 0.001) compared with those in the Control group (Fig. 5A). Moreover, there was no significant difference in SF-12 MCS scores between the Control group and the WIKAP group at M0 (*P  *= 0.438) and M3 (*P  *< 0.056) (Fig. 5B). However, the SF-12 MCS scores were significantly higher in the WIKAP group at M6 (*P  *= 0.002), M9 (*P  *< 0.001), and M12 (*P  *< 0.001) post-intervention compared to those in the Control group (Fig. 5B). Furthermore, the changes in SF-12 PSC (M12-M0) score and SF-12 MCS score (M12-M0) were much higher in the WIKAP group than those in the Control group after the 12-month intervention (*P  *< 0.001, respectively, as shown in Fig. 5C and D).

### The effect of web-based IKAP nursing on MACCE occurrence

**Fig. 6:**
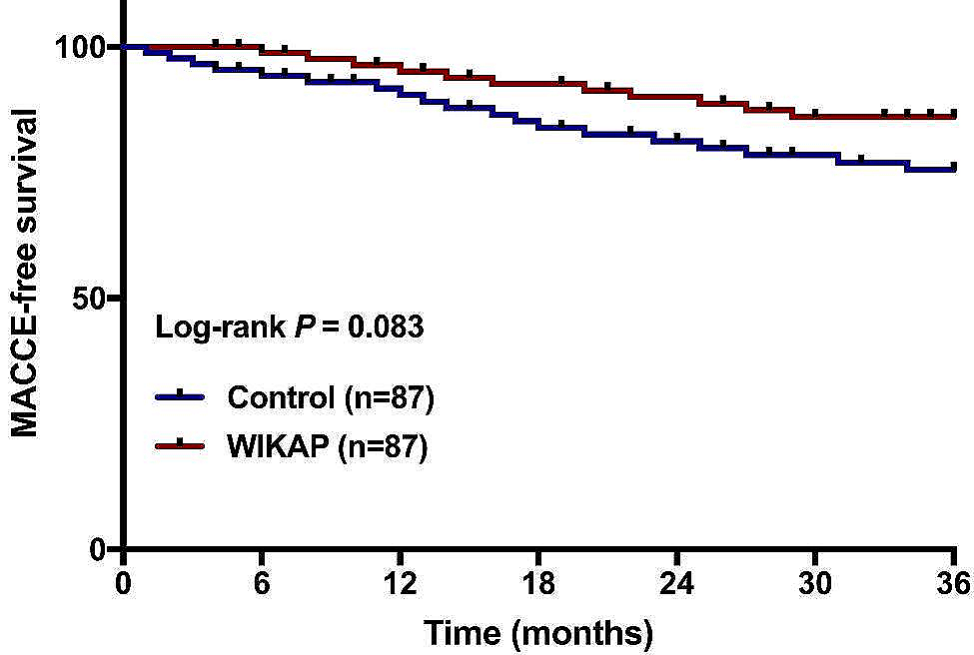
Kaplan-Meier curve analysis of MACCE-free survival in CAD patients with CABG post-operation between the Control and WIKAP groups during a 36-month follow-up. *P* < 0.05 was considered statistically significant

The incidence of MACCE was monitored and recorded throughout the entire 36-month follow-up period for all participants in this study. The findings revealed that the incidence of MACCE was observed in both groups (Table 3), and the occurrence rate for each MACCE, including myocardial infarction (*P  *= 0.700), stroke & thromboembolism (*P  *= 0.469), repeat revascularization (*P  *= 0.350), and death (*P  *= 0.312) showed no significant difference between the WIKAP group and the Control group (Table 3). The total number of MACCE occurrences in the WIKAP group was 11 cases (12.64%), which was relatively lower than that in the Control group (19 cases, 21.84%), but without a significant difference (*P  *= 0.108, Table 3). Meanwhile, we also found that although the MACCE-free survival curve during the 36-month follow-up between the Control group and WIKAP group showed no statistical significance (*P* = 0.083, Fig. 6), the trend of MACCE-free survival in the WIKAP group was slightly longer than that in the Control group (Fig. 6).

**Table 3:** The comparison of MACCE occurrence in CAD patients after CABG surgery between the two groups during a 36-month follow-up

Items	Control group (*n* = 87)	WIKAP group (*n* = 87)	χ^2^	*P*
Myocardial infarction, no (%)	4 (4.60)	3 (3.45)	0.150	0.700
	5 (5.75)	3 (3.45)	0.527	0.469
Repeat revascularization, no (%)	7 (8.05)	4 (4.60)	0.881	0.350
Cardiac death^a^, no (%)	3 (3.45)	1 (1.15)	1.024	0.312
Total MACCE, no (%)			2.578	0.108

**Notes**: Statistical analysis was calculated by Student’s *t*-test. CABG, coronary artery bypass grafting; CAD, coronary heart disease; MACCE, major adverse cardiac and cerebrovascular events; WIKAP, web-based Information-Knowledge-Attitude-Practice. ^a^Cardiac death is defined as death caused by heart failure or acute coronary syndrome

### Cox proportional hazards regression analysis for MACCE occurrence

As shown in Table 4, the univariate Cox regression analysis revealed that WIKAP (vs. Control) was not associated with the occurrence of MACCE (*P* = 0.089, HR = 0.525, 95% CI 0.250–1.104). However, age (*P* = 0.003, HR = 3.058, 95% CI 1.455–6.427), NYHA classification (III) (*P* = 0.014, HR = 2.492, 95% CI 1.200-5.173), more lesion vessels (*P* = 0.002, HR = 3.224, 95% CI 1.524–6.776), hypertension (*P* = 0.004, HR = 3.711, 95% CI 1.517–9.080), and diabetes (*P* = 0.005, HR = 2.820, 95% CI 1.378–5.772) were identified as risk factors for the occurrence of MACCE in CAD patients (Table 4). According to the results of the univariate Cox regression model, the risk factors with *P* < 0.05 were further analyzed in the multivariate Cox proportional hazard regression analysis. The analysis revealed that age (*P* = 0.043, HR = 2.243, 95% CI 1.027–4.897), NYHA classification (III) (*P* = 0.006, HR = 2.763, 95% CI 1.329–5.743), presence of more lesion vessels (*P* = 0.012, HR = 3.185, 95% CI 1.293–7.846), hypertension (*P* = 0.026, HR = 2.431, 95% CI 1.112–5.317), and diabetes (*P* = 0.024, HR = 2.356, 95% CI 1.119–4.961) were identified as independent risk factors for the occurrence of MACCE in CAD patients (Table 4).

**Table 4:** Cox proportional hazards regression analysis of variables associated with the occurrence of MACCE among CAD patients after CABG surgery

	Univariate analysis			Multivariate analysis		
Variables	HR	95% CI	*P*	HR	95% CI	*P*
Group (Control vs. WIKAP)	0.525	0.250–1.104	0.089			
Age (≥ 65 years)	3.058	1.455–6.427	**0.003**	2.243	1.027–4.897	**0.043**
Age (≥ 25 kg/m^2^)	0.907	0.424–1.937	0.801			
Male	0.705	0.336–1.483	0.357			
Higher education level	0.893	0.425–1.877	0.765			
Single/windowed/divorced	1.233	0.564–2.691	0.600			
Employed	0.827	0.398–1.718	0.611			
Lower annual household income	0.893	0.393–2.081	0.793			
Rural	0.920	0.447–1.893	0.820			
NYHA classification (III)	2.492	1.200-5.173	**0.014**	2.763	1.329–5.743	**0.006**
More lesion vessels	3.224	1.524–6.776	**0.002**	3.185	1.293–7.846	**0.012**
Hypertension	3.711	1.517–9.080	**0.004**	2.431	1.112–5.317	**0.026**
Hyperlipidemia	0.760	0.366–1.578	0.462			
Diabetes	2.820	1.378–5.772	**0.005**	2.356	1.119–4.961	**0.024**
Smoker history	2.005	0.939–4.284	0.072			
Family history of CAD	1.758	0.782–3.949	0.172			
LVEF (&lt; 50%)	1.609	0.775–3.341	0.202			

**Notes.** BMI, body mass index; CABG, coronary artery bypass grafting; CAD, coronary heart disease; CI, confidence interval; HR, hazard ratio; MACCE, major adverse cardiac and cerebrovascular events; LVEF, left ventricular ejection fraction; NYHA, New York heart association; WIKAP, web-based Information-Knowledge-Attitude-Practice. *P* < 0.05 was considered significant

## Discussion

To the best of our knowledge, this is the first study to examine the effects of the WIKAP intervention on the health outcomes of CAD patients who have undergone CABG surgery in China. In the present study, a novel WIKAP continuous nursing program was designed and implemented for CAD patients after CABG surgery. We found that a web-based continuous intervention service called WIKAP, implemented through the Internet, could significantly reduce anxiety and depression in CAD patients. Additionally, it remarkably improved medication compliance and QoL during the 12-month intervention follow-up. These findings suggested that WIKAP continuous intervention had the potential to supplement the deficiencies and limitations of conventional routine nursing to some extent. However, the implementation of WIKAP showed no significant advantage in reducing MACCE occurrence compared to the control group during the entire 36-month follow-up period, which requires further investigation.

It is worth mentioning that the implementation of novel continuous nursing models has been found to yield positive results in the rehabilitation of CAD patients undergoing CABG. For instance, Jin et al. have suggested that family nursing combined with the network could effectively improve the prognosis and QoL in patients who underwent CABG [21]. Ma et al. have further stated that adverse emotions, QoL, and CAD risk could be obviously improved in patients who have undergone CABG surgery by utilizing a WeChat-based nursing program [15]. Nowadays, with the widespread availability of the Internet to the majority of the population in China, various social software platforms such as QQ and WeChat (with approximately one billion users) have become the most popular communication tools for Chinese people to exchange electronic information with each other [22]. Therefore, targeted nursing interventions that take advantage of the Internet have been confirmed to efficiently assist CAD patients and their caregivers in gaining a comprehensive understanding of the disease’s pathogenesis, progression, complications, and risk factors [7, 23]. However, the impact of the WIKAP continuous nursing intervention on the rehabilitation of CAD patients following CABG operation remains largely unknown.

According to the IKAP theory, patients who receive proper and effective disease-related knowledge based on their individual needs are more likely to enhance their self-care abilities, boost their confidence, and ultimately translate this health-related knowledge into actual practice to improve their own healthy behaviors [10, 24]. In regard to CAD patients who have undergone CABG, prior researchers have suggested that the establishment of a close nurse-patient interaction has been confirmed to play a critical role in enhancing their compliance and restoring their confidence after being discharged [25]. Consistently, in the current study, the medical compliance index MMAS-8 was obviously increased in the WIKAP group over the 12-month intervention period, particularly at M6, M9, and M12 time points, suggesting the positive impact of the WIKAP continuous nursing program on improving patient medication compliance after CABG operation. These findings might be explained by the fact that the instant messaging functions of Internet social media platforms like WeChat and QQ allowed nursing workers to promptly identify potential risk factors that influenced patients’ health-related behaviors based on the WIKAP theory. This enabled them to solve problems in real-time, greatly benefiting patients and further enhancing their motivation to engage in the WIKAP nursing program. Similarly, previous literature has also emphasized that the IKAP theory could effectively encourage patients to take a more active role in disease management and consciously learn useful self-care skills to solve their own problems, as compared to the conventional routine nursing mode [10].

Most patients who undergo CABG are inevitably going to experience feelings of tension and fear, both before and after surgery [26]. Nevertheless, during the short-term hospitalization, it is impossible for patients to acquire all the knowledge and skills necessary for disease management solely from the nursing staff. Accordingly, once the sudden gap in care arises after discharge, many patients and their caregivers often feel helpless in dealing with the disease-related challenges they encounter during the extended period of rehabilitation, particularly for patients living in rural areas with limited access to medical facilities [27]. Therefore, insufficient knowledge and lack of professional nursing guidance always result in significant psychological burdens for most patients with CAD undergoing CABG surgery, which are even correlated with poor QoL [28]. In our study, we utilized the WIKAP nursing intervention through Internet dissemination. The responding nurse encouraged patients to actively share and exchange their experiences about CABG rehabilitation in certain QQ and WeChat groups. Once patients with severe anxiety and negative emotions were identified, they would receive private psychological counseling from our professional therapist. The goal of this counseling is to help these patients overcome their negative mental feelings and regain their confidence, using the WIKAP theory as a guide. Accordingly, our findings showed that the anxiety and depression indexes, HADS-A and HADS-D scores, were gradually reduced in the WIKAP group. A significant difference was observed between the WIKAP and the Control groups at M9 and M12 post-intervention. Additionally, the QoL indicators SF-12 PCS and SF-12 MCS scores were apparently elevated in the WIKAP group compared to those corresponding scores in the Control group at M6, M9, and M12 post-intervention, suggesting a significant improvement in physical activity and mental status among patients who received WIKAP continuous nursing intervention.

Accumulating evidence has demonstrated that the occurrence of MACCE was closely associated with impaired physical function and psychological status in patients with CAD after CABG surgery. This association has been shown to have a negative impact on prognosis and increase mortality rates [29]. In the present study, considering that the implementation of WIKAP continuous nursing intervention had a positive impact on patients by enhancing their psychological well-being and improve their QoL, we assumed that WIKAP continuous nursing might influence the incidence of MACCE in patients with CAD following CABG surgery. Therefore, the incidence rate of MACCE was compared between the WIKAP and the Control groups for another 2-year follow-up. Here, a prolonged trend of MACCE-free survival was observed in patients after CABG in the WIKAP group compared with that in the Control group. The possible explanations were that the responding nurses in the WIKAP program consistently monitored the participants’ daily behavior through web communications and provided guidance on how to cope with risky situations, thereby contributed to a reduction of occurrence of MACCE in CAD patients. Nevertheless, no significant difference in MACCE-free survival was found between the Control and WIKAP groups. Further univariate and multivariate Cox regression analysis also confirmed that the nursing intervention (Control vs. WIKAP) was not associated with the occurrence of MACCE. This might be due to the low statistical power resulting from the limited sample size involved in the current study. Therefore, it is imperative to conduct further research with a larger sample size to verify these findings. Notably, previous literature has emphasized the crucial association of physiological and pathological indicators such as age, hyperlipidemia, hypertension, and diabetes with the occurrence of MACCE among patients with CAD [30, 31]. Similarly, in our study, we identified several clinical indexes including age, NYHA classification (III) of cardiac function, more lesion vessels, hypertension, and diabetes, as independent risk factors for the occurrence of MACCE in post-operative CAD patients. Thus, compared to these commonly risky indexes, we hypothesized that the WIKAP intervention alone might be insufficient to demonstrate significant effects on the occurrence of MACCE in CAD patients who underwent CABG surgery. Other clinically innovative therapeutic strategies should also be considered for the prevention of MACCE [32, 33].

### Limitations

We had to admit that there were some limitations in our research. First, since the small sample size was limited to participants from only one hospital, there was a potential for selection bias. Therefore, it is necessary to conduct large-scale and multi-locale investigations to validate our findings. Second, the assessment of depressive and anxious symptoms was solely assessed using the HADS scale. To enhance the validity of our findings, additional assessment methods for depression and anxiety would be required. Third, only a 12-month period of nursing care was implemented in this investigation. No analysis was conducted on the long-term effects of psychosocial indicators during the additional 24-month follow-up. Therefore, a more randomized controlled study with a long-term intervention follow-up period is needed for further validation. Fourth, in the MACCE analysis, missing data for follow-up loss were censored, and it was acknowledged that potential bias was unavoidable. Hence, further investigations are still needed to validate the effect of WIKAP continuous nursing intervention on the outcomes of CAD patients with CABG post-operation.

## Conclusion

WIKAP continuous intervention exhibited positive effects in caring for CAD patients with CABG post-operation. It made significant efforts to meet the patients’ needs in a fast, flexible, and cost-effective manner by remarkably alleviating patients’ depression and anxiety, promoting medical compliance, and improving health-related QoL. Furthermore, it showed a prolonged trend of MACCE-free survival among patients after CABG surgery. These findings suggest that the WIKAP nursing intervention has promise as a strategy for post-operative CABG management.

## Data Availability

The datasets used for analyzed in this study are available from the corresponding author on reasonable request.

## Abbreviations

BMI: Body mass index
CAD: Coronary heart disease
CABG: Coronary artery bypass grafting
CVD: Cardiovascular diseases
CI: Confidence interval
HADS-A: Hospital Anxiety and Depression Scale-Anxiety
HADS-D: Hospital Anxiety and Depression Scale-Depression
HR: Hazard radio
ITT: Intention-to-treat
LVEF: Left ventricular ejection fraction
MACCE: Major adverse cardiac and cerebrovascular events
MMAS-8: Morishy’s medication adherence scale
NYHA: New York heart association; SD, standard deviation
SF-12 PCS: 12-Item Short-Form Health Survey -Physical Component Summary
SF-12 MCS: 12-Item Short-Form Health Survey -Mental Component Summary
WIKAP: Web-based Information-Knowledge-Attitude-Practice
